# Effects of miR-107 on Breast Cancer Cell Growth and Death via Regulation of the PTEN/AKT Signaling Pathway

**DOI:** 10.1155/2023/1244067

**Published:** 2023-02-08

**Authors:** Hua Pan, Hongwei Peng, Yun Dai, Bin Han, Guangming Yang, Nian Jiang, Ping Zhou

**Affiliations:** ^1^Liyang People's Hospital, Liyang, China; ^2^Faculty of Medicine, University of Tsukuba, Tsukuba, Japan

## Abstract

**Objective:**

Investigate the influence of miR-107 on breast cancer cell growth and death through the PTEN/AKT signaling pathway.

**Method:**

As study subjects, the human breast cancer cell line MCF-7 and the normal breast cell line Hs 578Bst were chosen, and MCF-7 cells were, respectively, transfected with control miRNA and miR-107 inhibitor. CCK-8, flow cytometry, scratch assay, and Transwell assay were used to analyze the proliferation, apoptosis, and invasion, and in order to identify the proteins associated with apoptosis in each of the three categories, we used western blot analysis. Bcl-2, cleaved caspase-3, and cleaved caspase-9 expression, as well as PTEN/AKT signaling pathway-associated protein expression, are correlated.

**Result:**

The expression of miR-107 in MCF-7 cells was significantly greater than that in Hs 578Bst cells, with a *P* < 0.05 difference; compared to the blank and miRNA control groups, the miR-107 inhibitor group had a *P* < 0.05 difference. *P* < 0.05 showed a decrease in proliferation (42.52) but no difference in proliferation between the blank and miRNA control groups (*P* > 0.05); the miR-107 inhibitor group had higher apoptosis (38.96) with *P* < 0.05 than the blank group (4.85) and the miRNA control group (5.89); there was no difference in apoptosis between the blank and miRNA groups (*P* > 0.05). There was no significant difference between the blank group and the miRNA control group with *P* > 0.05; compared with the blank group, the miR-107 inhibitor group had a lower expression of Bcl-2 protein (0.18), in addition to the degraded paradigms (0.73) and caspase-9 protein concentrations (0.79), respectively.

**Conclusion:**

The PTEN/AKT signaling pathway may be regulated by miR-107 to limit breast cancer cell growth and increase apoptosis, which suggests that miR-107 may be exploited as a tumor marker for therapeutic therapy.

## 1. Introduction

One in every eight women will get cancer of the breast or its ducts over their lifetime [1]. This form of tumor has a bad prognosis because of its advanced spread. Many components and processes interact to cause the condition, but there is no definitive scientific conclusion [2]. Hair loss, nausea, vomiting, thrombosis, bone marrow suppression, and other adverse effects are common with treatments such as surgery, radiation, and chemotherapy. As a result, new anticancer medicines with reduced toxicity and side effects are urgently needed. A major function in cancer cell biology and pathology may be played by the miRNA class. Studies have demonstrated that miR-200 regulates a wide variety of malignant tumor cells through specific regulatory proteins [3, 4]. We also know that cell proliferation and death have an impact on cell viability, and clonality reflects both the capacity of cells to proliferate and their reliance on populations. Because of this, we should begin with cell proliferation and apoptosis in order to find the most effective treatments. Nonconservative miRNAs such as miR-107 are becoming more and more common. The EMT process of pancreatic cancer seems to be regulated by this protein, and its expression is aberrant in a variety of tumor tissues, including gastric cancer, according to certain research. Cancer cell proliferation, migration, and migration may all be affected by a decrease in miR-107 expression. The upregulation of miR-107 expression level has also been seen in breast cancer tissue [5, 6]. There is a tumor suppressor gene identified in recent years known as PTEN, which mediates AKT and other signaling pathways in the emergence and progression of many malignancies. By modulating the PTEN/AKT pathway, miRNAs have been demonstrated to have a positive impact on breast cancer progression [7, 8]. The PI3K/AKT pathway can be activated via PTEN by miR-107, which has been shown to reduce myocardial damage caused by sepsis as well as to affect the apoptosis of LPS-induced cardiomyocytes [9, 10]. Other studies have discovered long noncoding RNA in gastric cancer cells that can be targeted to regulate the PTEN/PI3K/AKT pathway. It is not known, however, whether miR-107 regulates this pathway and has an impact on breast cancer. It is thus necessary to investigate how miR-107 affects breast cancer cell proliferation and death via modulating the PTEN/AKT signaling pathway and its mechanism, which serves as a reliable guide for therapeutic therapy.

## 2. Components and Procedures

### 2.1. Equipment

Bcl-2 antibody, cleaved caspase-3 antibody, and cleaved caspase-9 antibody were purchased from Shanghai Sangon Co., Ltd.; BCA protein concentration detection kit, Annexin V-FITC apoptosis death detection kit, goat anti-mouse PTEN antibody, and goat immunoglobulin were bought from Guangzhou Ribo Bio Co., Ltd. miR-107 inhibitor was purchased from Guangzhou Ribo Bio Co., Ltd. Goat antibody was purchased from Guangzhou Ribo Bio Co., Ltd. Internal reference antibody for mouse p-AKT A PCR instrument, centrifuge (Dalong Xingchuang Experimental Instrument Co., Ltd.), electrophoresis instrument (United States Bio-Rad Company), flow cytometer (United States BD Company), and a microscope (Olympus, Japan) are all examples of instruments in this category that can be found in the United States.

### 2.2. Procedures

#### 2.2.1. Cell Culture

Cell lines Hs 578Bst and MCF-7 were grown in DMEM+10% FBS conditions. All of the cells were separated into three groups: the control group, the blanks group, and the miR-107 inhibitors group. For miR-107 and miRNA control transfection, add 100 microliters of Opti-MEM medium to two 1.5-mL centrifuge tubes, followed by 5 microliters of the transfection reagent. Mix them gently after 5 minutes at room temperature. Stand for another five minutes before adding to the culture medium for the cells. Six hours after transfection, the medium was changed to a fresh one.

#### 2.2.2. Detection of miR-107 Activity in Cell Lines via PCR

After centrifugation, the cells were washed with PBS, the total RNA was extracted according to the kit's instructions, cDNA was synthesized, and PCR detections were made using GAPDH as an internal reference. PCR amplification was performed for 40 cycles. Quantitative analysis was performed. miR-107 expression was detected. Primer: miR-107 upstream 5′-CATACTAGTGTCTTCTGGACAGGCTCTG-3′, downstream: 5′-CTTAAGCTTAGAATCTCTCACATACACAC-3′. GAPDH upstream: 5′-GCACCGTCAAGGCTGAGAAC-3′, downstream: 5′-TGGTGAAGACGCCAGTGGA-3′.

#### 2.2.3. Detection of Cell Proliferation by Using CCK-8

At a density of 810 3 cells per well, cells were plated on 96-well plates and transfected in groups. After 24 hours of transfection, add 10 L of CCK-8 reagent to each well and measure absorbance levels by using a spectrophotometer at 450 nm wavelength after a 1-hour shaking.

#### 2.2.4. Detection of Apoptosis by Flow Cytometry

Collect the cells, firstly adjust the cell concentration with 10% PBS solution, then wash twice with washing solution, then add trypsin solution to digest the cells, then suspend, then centrifuge in a centrifuge. Culture cells in a 37°C, 5% CO_2_ constant temperature incubator after removing cell debris. The images were collected at 0 h and 48 h under the microscope. Mobility as a percentage = (0-hour scratch distance − 24-hour scratch distance)/0-hour scratch distance 100%.

#### 2.2.5. Scratch Test to Detect Cell Migration

Until the cells reached 80% to 90% confluence, they were collected and allowed to continue to develop in a monolayer adherent form. Scrub the cells with a perpendicular pipette tip and then wash the cells three times with PBS to confirm that the scratch width is constant. Culture cells in a 37°C, 5% CO_2_ constant temperature incubator after removing cell debris. The images were collected at 0 h and 48 h under the microscope. Percentage of mobility/hour = (0-hour scratch distance − 24-hour scratch distance)/0-hour scratch distance 100%.

#### 2.2.6. Transwell Assay to Detect Cell Invasion

Centrifugation and trypsin digestion followed the collection of cells, which were then washed one time with PBS following centrifugation. The first step was to adjust the cell density to 510 5 cells/mL. Matrigel-coated Transwell incubator was used to seed cells, and the chamber was put on a cell culture plate containing 10% FBS media. Cells were then cultivated for 24 hours at 37°C CO_2_. Matrigel and cells were cleaned off with a cotton swab and preserved in a 4 percent formaldehyde solution for 10 minutes after the Transwell chamber was removed. A 0.1 percent crystal violet solution was used for 10 minutes to stain the cells that had settled to the chamber's bottom before the cells were washed and examined under a microscope.

#### 2.2.7. Detection of Cellular Protein Expression via Immunoprecipitation

You must collect the transfected cells before centrifuging them at 12,000 r/min for 5 minutes with the supernatant to evaluate total protein concentration. Fill the gel to the brim with electrophoresis fluid and then reload the sample. Afterwards, the electrophoresis and membrane transfer were followed by TBS soaking, shaking, and blocking for an hour, and the addition of Bcl-2, cleaved caspase-3, cleaved caspase-9, PTEN, and p-AKT antibodies were added (1 : 1000). Secondary antibody (1 : 5000) was incubated overnight at 4°C, GAPDH was used as an internal reference after developing solution was added for color development, and the gel was photographed throughout the reaction, which lasted 30 minutes at 37 degrees Celsius. Finally, the gel is subjected to a developing solution in order to develop its color.

### 2.3. Statistical Methods

The statistical analysis of data was performed using SPSS 21.0 software. Mean standard(s) and independent samples(s) were used to represent measurement data. *P*0.05 was deemed statistically significant for comparisons between two groups using the *t* test, comparisons between multiple groups using the one-way analysis of variance, and comparisons between two groups using the LSD-t test for multiple comparisons.

## 3. Results

**Figure 1:**
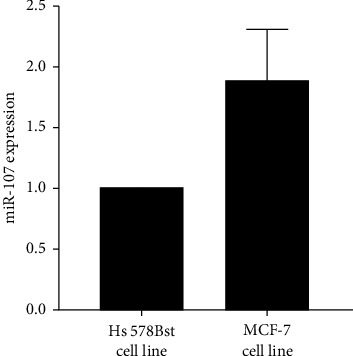
Comparison of miR-107 expressions in cells of each group.

Compared to the Hs 578Bst cell line, the expression of miR-107 in the MCF-7 cell line was greater with a *P* 0.05, as shown in Table 1 and Figure 1 (Table 1).

**Figure 2:**
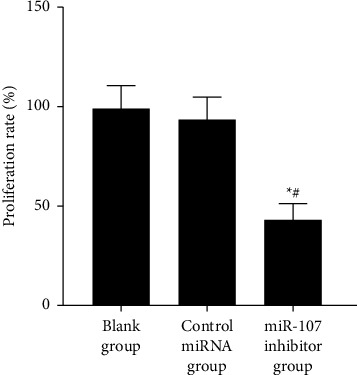
Comparing cell proliferation rates.

On the other hand, there was no significant difference found between the blank and the miRNA control groups with *P* > 0.05 in the proliferation rate of the miR-107 inhibitor group.  Table 2 and Figure 2, respectively, indicate this impact of inhibition on cell proliferation.

**Figure 3:**
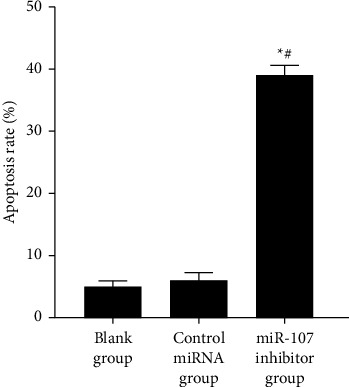
Comparison of each group's apoptosis rates.

**Figure 4:**
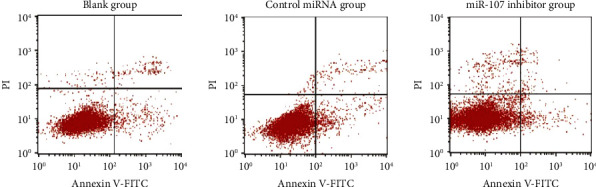
Flow experiment results.

miR-107 inhibitors had a greater apoptotic rate than the blank and miRNA control groups, with *P* 0.05; there was no significant difference between the blank group and the miRNA control group Difference with *P* > 0.05, as shown in Table 3 and Figures 3 and 4.

**Figure 5:**
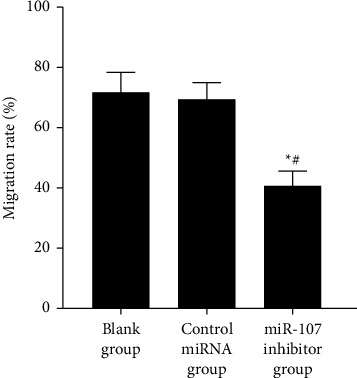
Comparison of the mobility of each group.

**Figure 6:**
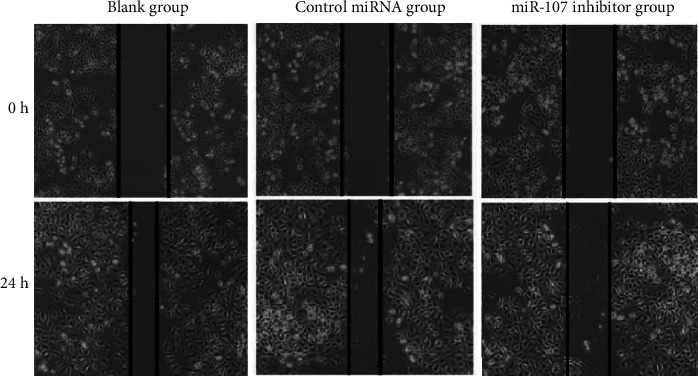
Scratch test results (40×).

miR-107 inhibition has an impact on cell migration: Figures 5 and 6 reveal that the miR-107 inhibitor group migrated less than the blank or control miRNA groups (*P* 0.05), although the migration rates of the two other groups (*P* > 0.05) were not significantly different (Table 4, Figures 5 and 6).

**Figure 7:**
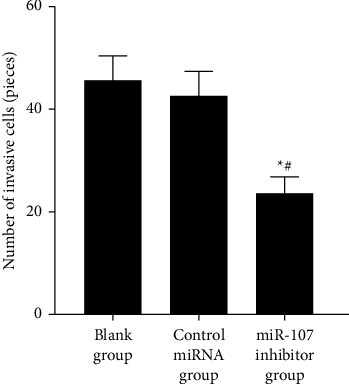
Invasive cell count comparison.

**Figure 8:**
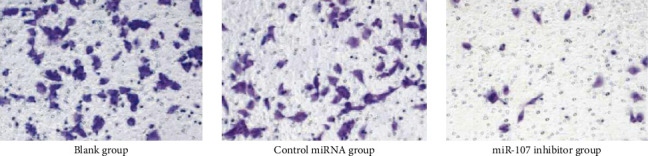
Transwell experiment results (200×).

Cell invasion is reduced when miR-107 is inhibited. As shown in Table 5 and Figures 7 and 8, fewer invasive cells were found in the miR-107 inhibitor group than in the control or blank groups (*P* 0.05). There was no difference between these two groups (*P* > 0.05) in the number of invasive cells.

**Figure 9:**
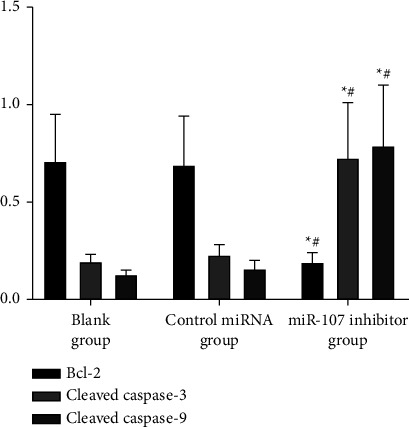
Comparison of apoptosis protein expression in each group.

**Figure 10:**
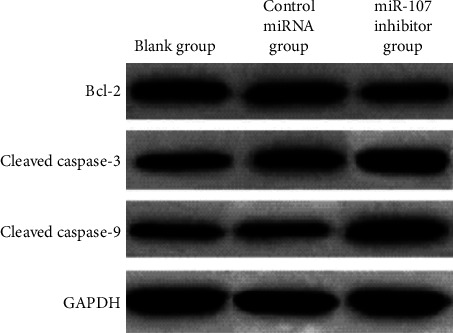
Comparison of apoptosis protein expression in each group.

miR-107 inhibition reduced the expression of Bcl-2 protein and increased the expressions of cleaved caspase-3 and cleaved caspase-9 proteins, compared to the blank group, While the miRNA control group and the miRNA blank group showed no change. The data are summarized in Table 6 and Figures 9 and 10.

**Figure 11:**
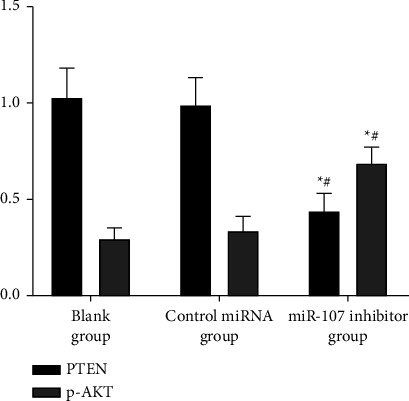
Comparison of PTEN and p-AKT protein expression in cells of each group.

**Figure 12:**
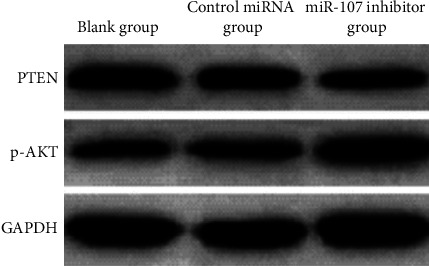
Comparison of PTEN and p-AKT protein expression in cells of each group.

**Figure 13:**
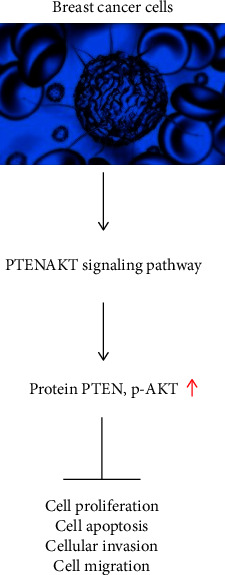
Action diagram of the signaling pathway.

PTEN and p-AKT protein expression were decreased in the miR-107 inhibitor group compared to the blank group and the miRNA control group with *P* 0.05; there was no significant difference between the blank group and the miRNA control group with *P* > 0.05, as shown in Table 7 and Figures 11–13.

## 4. Discussion

Breast cancer is becoming more common every year. It has become a major issue in clinical therapy because of its invasiveness and high recurrence rate [11]. Tumor cells are regulated by miRNAs, which have a significant impact on their biology. According to research, tumor tissue miRNA expression levels are closely linked to the severity and prognosis of cancer [12, 13]. Prostate and liver cancer cells may be inhibited by miR-107, according to previous studies. The STK33/ERK signaling pathway, for example, may regulate lung cancer cells' malignant biological activity [14]. Downregulating miR-107 expression has previously been shown to have an effect on cell proliferation and migration in multiple cancer cells, whereas the upregulation of this miRNA in breast cancer tissues has been linked to lymphatic metastasis. However, it is unclear if miR-107 plays a regulatory function in the development of breast cancer [6, 15, 16].

To begin, the expression of miR-107 in normal and cancerous human breast cell lines was evaluated in this work. According to the results, the expression of miR-107 was shown to be significantly higher in human breast cancer cell lines compared to normal breast cell lines. miR-107 is highly expressed in breast cancer, which suggests that suppressing miR-107 may have a tumor-suppressive impact on breast cancer. As we all know, the most typical and essential feature of cancer cells is proliferation, so controlling this feature can effectively prevent the occurrence of diseases. In addition, the most efficient way to maintain the basic activity of cells is apoptosis. Because it can participate in the body's ability to recognize and deal with virus-infected cells in the body, and by regulating and improving damaged cells, it can inhibit the occurrence of cancer, and it is considered to be the key research direction of clinical treatment. In this study, the proliferation of cells in each group was examined, and it was discovered that the miR-107 inhibitor group's proliferation rate was lower than that of the blank group and that of the miRNA control group but that the proliferation rates of the blank group and the miRNA control group were not significantly different. This suggests that miR-107 inhibition may reduce breast cancer cell growth. When it comes to cell death, miR-107 inhibitors had a greater rate than miRNA control groups, but there was no significant difference in cell death rates between blank groups and miRNA control groups, according to this study. This suggests that miR-107 inhibition affects breast cancer cell growth and apoptosis. Breast cancer cells may be inhibited from autophagy, proliferation, and migration by HMGB1 [17]; in addition, several research studies have discovered that miR-107 controls PTX sensitivity via Wnt/-catenin signaling pathway, which targets TPD52 [18–20], [17, 18, 18–20]. The most dangerous and life-threatening behavior of cancer cells is invasion and metastasis. It is not only a behavior with a more complex process but also interfered by many kinds of factors. For example, the expression of hydrolyzed protein peptide chains or the regulation of signaling pathways may affect biological behavior. Therefore, this is also one of the key research directions of clinical treatment, which can inhibit the disease by controlling or blocking these steps. Therefore, for further verification, cell invasion and migration were observed in this study. miR-107 inhibitors had a lower migration rate than the blank and control miRNA groups, but there was no significant difference in migration rate between the blank and control miRNA groups. The number of invasive cells was lower in the miR-107 inhibitor group than in the blank and control miRNA groups, but there was no significant difference between the b groups. Furthermore, this shows that inhibiting miR-107 has a considerable inhibitory impact on it; confirming those findings, researchers anticipate that by changing tumor cells into cells that support parenchymal cells in the completion of organ tasks, miR-107 will boost cell invasion and metastasis in breast cancer cell lines [19, 20]. In addition, it is well known that in the biological behavior of cells, there is an indicator that can significantly monitor the degree of cell damage, namely apoptosis. Basic research shows that PTEN can promote apoptosis. PTEN antagonizes the activity of the Akt pathway, promotes the release of reactive oxygen species, inhibits the activity of NF-*κ*B, and finally induces apoptosis. There are other studies showing that PTEN can also induce cell death by activating proapoptotic proteins [21, 22]. However, whether it can play this role in breast cancer cells needs further verification. Therefore, in this study, we mainly analyzed apoptosis-related proteins. The results showed that compared with the blank group and the control miRNA group, the expression of Bcl-2 protein in the miR-107 inhibitor group was lower. There was no difference between the blank group and the miRNA control group in cleaved caspase-3 and -9 protein expression. Inhibiting miR-107 inhibited breast cancer cell growth and apoptosis.

Studies have shown that PTEN is an enzyme that has exact specifications related to substrates and is a negative regulator of this system. This was discovered by ridiculing particular tyrosine proteins or by lowering the activity of the pathway that involves PTEN, PI3K, and AKT, which may decrease the tumor's destructive progress. Previous studies have demonstrated that miRNAs may activate this pathway and enhance proliferation, migration, and invasion in breast carcinomas that lack PTEN [7, 23]. The tumor suppressor effect of PTEN is mainly reflected in its participation in multiple signal transduction pathways such as PTEN/PI3K/Akt, PTEN/ERK, and PTEN/FAK/P130cas, and suppressing tumor cell invasion and metastasis, halting cell cycle, and preventing tumor angiogenesis are only a few of the many roles it performs [24, 25]. As a result of this research, the miR-107 inhibitor group had lower PTEN protein expression and greater p-AKT protein expression than the blank group and the miRNA control group; there was no significant difference between the blank group and the miRNA control group. This suggests that the PTEN/AKT signaling pathway may be inhibited by inhibiting miR-107 in breast cancer cells.

In conclusion, breast cancer cells express high miR-107, and inhibiting miR-107 can inhibit the proliferation of breast cancer cells, promote their apoptotic response, and affect cell migration and invasion. It may be achieved by regulating the PTEN/AKT signaling pathway. Using miR-107 as an early diagnosis and a novel targeted drug for the therapeutic therapy of this illness is possible, according to this research. This study was limited to the cellular level since animal studies have not been conducted; thus further research is required to determine the mechanism of action.

## Figures and Tables

**Table 1:** Comparison of miR-107 expressions in cells of each group ($\bar{\chi }$ ± *s*).

Grouping	*n*	miR-107
Hs 578Bst cell line	6	1.00 ± 0.00
MCF-7 cell line	6	1.89 ± 0.42
*t*		5.212
*P*		0.003

**Table 2:** Comparison of cell proliferation rates in each group ($\bar{\chi }$ ± *s*).

Grouping	*n*	Proliferation rate (%)
Blank group	6	98.52 ± 12.05
Control miRNA group	6	93.14 ± 11.53
miR-107 inhibitor group	6	42.51 ± 8.69^∗^^#^
*F*		48.593
*P*		<0.001

*Note.* ^∗^ *P* 0.05 in comparison to the blank group; ^#^*P* 0.05 in comparison to the control miRNA group.

**Table 3:** Comparison of each group's apoptosis rates ($\bar{\chi }$ ± *s*).

Grouping	*n*	Apoptosis rate (%)
Blank group	6	4.85 ± 1.05
Control miRNA group	6	5.89 ± 1.32
miR-107 inhibitor group	6	38.96 ± 1.63^∗^^#^
*F*		1229.819
*P*		<0.001

*Note.* ^∗^ *P* 0.05 in comparison to the blank group; ^#^*P* 0.05 in comparison to the control miRNA group.

**Table 4:** Comparison of cell migration rates in each group ($\bar{\chi }$ ± *s*).

Grouping	*n*	Mobility (%)
Blank group	6	71.52 ± 6.85
Control miRNA group	6	69.23 ± 5.73
miR-107 inhibitor group	6	40.36 ± 5.21^∗^^#^
*F*		50.835
*P*		<0.001

*Note.* ^∗^ *P* 0.05 in comparison to the blank group; ^#^*P* 0.05 in comparison to the control miRNA group.

**Table 5:** Comparison of the number of invasive cells in each group ($\bar{\chi }$ ± *s*).

Grouping	*n*	Number of invasive cells (pcs)
Blank group	6	45.50 ± 4.93
Control miRNA group	6	42.50 ± 4.85
miR-107 inhibitor group	6	23.50 ± 3.39^∗^^#^
*F*		43.204
*P*		<0.001

*Note.* Compared with the blank group, ^∗^*P* < 0.05; compared with the control miRNA group, ^#^*P* < 0.05.

**Table 6:** Comparison of each group's apoptotic protein expression ($\bar{\chi }$ ± *s*).

Grouping	*n*	Bcl-2	Cleaved caspase-3	Cleaved caspase-9
Blank group	6	0.70 ± 0.25	0.20 ± 0.03	0.13 ± 0.02
Control miRNA group	6	0.68 ± 0.26	0.23 ± 0.05	0.16 ± 0.04
miR-107 inhibitor group	6	0.18 ± 0.06^∗^^#^	0.73 ± 0.28^∗^^#^	0.79 ± 0.31^∗^^#^
*F*		11.817	19.442	25.852
*P*		0.001	<0.001	<0.001

*Note.* ^∗^ *P* 0.05 in comparison to the blank group; ^#^*P* 0.05 in comparison to the control miRNA group.

**Table 7:** Comparison of PTEN and p-AKT secretion of proteins in the cells of each category ($\bar{\chi }$ ± *s*).

Grouping	*n*	PTEN	p-AKT
Blank group	6	1.02 ± 0.16	0.30 ± 0.05
Control miRNA group	6	0.98 ± 0.15	0.34 ± 0.07
miR-107 inhibitor group	6	0.43 ± 0.10^∗^^#^	0.69 ± 0.08^∗^^#^
*F*		33.197	62.113
*P*		<0.001	<0.001

*Note.* ^∗^ *P* 0.05 in comparison to the blank group; ^#^*P* 0.05 in comparison to the control miRNA group.

## Data Availability

The labeled data set used to support the findings of this study is available from the corresponding author upon request.
